# The association between dietary phytochemical index with depression and quality of life in iranian adolescent girls

**DOI:** 10.1186/s13030-022-00234-5

**Published:** 2022-02-02

**Authors:** Abbas Ali Sangouni, Azam Ahmadi Vasmehjani, Majid Ghayour-Mobarhan, Gordon A. Ferns, Sayyed Saeid Khayyatzadeh

**Affiliations:** 1 Department of Nutrition, School of Public Health, Shahid Sadoughi University of Medical Sciences, 8914715645 Yazd, Iran; 2 Nutrition and Food Security Research Center, Shahid Sadoughi University of Medical Sciences, Yazd, Iran; 3 Metabolic syndrome Research Center, Mashhad University of Medical Sciences, Mashhad, Iran; 4 Brighton & Sussex Medical School, Division of Medical Education, BN1 9PH Falmer, Brighton, Sussex, UK

**Keywords:** Depression, Quality of life, Phytochemical, Diet, Adolescents

## Abstract

**Background:**

There is increasing evidence that the dietary intake of phytochemicals is inversely associated with severity of depression and positively associated with quality of life (QoL). The present study investigated the relationship between dietary phytochemical index (DPI) with depression and QoL scores in Iranian adolescent girls.

**Methods:**

A total of 733 adolescent girls from Mashhad and Sabzevar cities in northeastern Iran were entered into this cross-sectional study. Assessment of depression and QoL was performed utilizing the Beck Depression Inventory (BDI) and SF-12v2 questionnaire, respectively. Assessment of dietary intake was undertaken by a qualified dietitian, using a validated food-frequency questionnaire (FFQ) containing 168 food items. To explore the association between DPI with QoL and depression, logistic regression was used in crude and adjusted models.

**Results:**

The participants in the fourth quartile of DPI compared with the first quartile had a 50% lower odds of depression (OR: 0.50; 95% CI: 0.30-0.84, *P* = 0.009) This relation remained significant in all adjusted models. The adolescents in highest quartile of DPI compared with the first quartile had 38% lower odds of poor QoL (OR: 0.62; 95% CI: 0.41-0.94, *P* = 0.02). This association remained significant in adjusted models I and II, but not after adjusting for all confounding variables (OR: 0.67; 95% CI: 0.43-1.02, *P* = 0.06) (Model III).

**Conclusions:**

DPI was inversely associated with risk of depression. The association between DPI score and QoL remained unclear. Further prospective and interventional studies are required.

## Introduction

The prevalence of depression is increasing, and due to its socioeconomic costs has become a major challenge for public health globally [1, 2]. The estimated prevalence of depression is between 11.1 and 14.6%, and is higher during adolescence [3, 4]. Females are more likely to develop depression than males [4]. The estimated prevalence of depression in Iranian children and adolescents is 43.5% [4]. It is a main cause of teenage suicide [5, 6]. The symptoms of depression are depressed mood and anhedonia [7]. It has been confirmed that obesity, oxidative stress, and inflammation are contributors to the development of depression [8–11]. Quality of life (QoL), which is defined as “the perception of individuals about their position in life in the context of the culture and value systems which is related to their goals, expectations, standards and concerns”, is negatively affected by depression [12–14].

The investigations demonstrated the association of the quality of diet with depression [15, 16]. Adherence to the healthy dietary patterns containing high amount of vegetables, fruits, legumes and whole grains can exert a therapeutic effect on mental disorders, in particular depression [16, 17]. Phytochemicals such as phenolic acids, carotenoids, terpenoids, organosulfur compounds, and phytosterols are the antioxidant compounds of vegetables, fruits, whole grains, nuts and legumes [18]. The experimental studies have indicated the antidepressant activity of phytochemicals [19–21]. In addition, phytochemicals have beneficial effects on determinants of depression such as abdominal obesity, oxidative stress and inflammation [22–25]. Dietary phytochemical index (DPI), which is a scoring tool, can estimate the level of phytochemicals intake from diet [26]. Only one study evaluated the association between DPI and depression among adults, and found an inverse relationship between DPI scores and risk of depression [27]. To our knowledge, the association between DPI scores and QoL has not been evaluated. Therefore, we designed a cross-sectional study to investigate the association between scores of DPI with depression and QoL in adolescent females.

## Methods and materials

### Study population

**Fig. 1:**
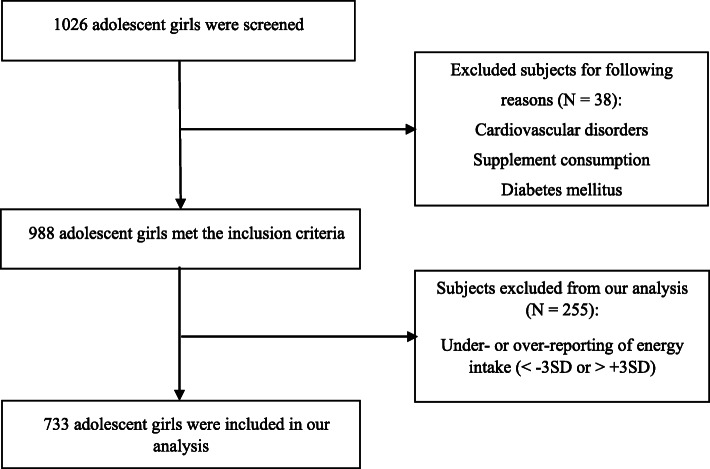
Flowchart of the data collection process of study

Utilizing a random cluster sampling method, a total of 1026 adolescent girls (aged 12-18 years) were selected from several schools of Mashhad and Sabzevar cities in northeastern Iran. 38 subjects were excluded, and 988 subjects were included in this cross-sectional study. In addition, 255 subjects were excluded from our analysis due to under-reporting of energy intake or over-reporting of energy intake, and 733 adolescent girls were included in our analysis (Fig. 1). The exclusion criteria were: a history of autoimmune diseases, metabolic bone disease, all types of cancer, cardiovascular disorders, diabetes mellitus, hepatic or renal failure, malabsorption, thyroid, parathyroid, adrenal diseases, anorexia or bulimia nervosa, parental dissatisfaction, taking anti-inflammatory, anti-depressant, antidiabetic, or anti-obesity medications, consuming vitamin D or calcium supplement, and hormone therapy within the last 6 months. Before the beginning of the study, the subjects completed written informed consent, and the study was approved by the ethical committee of Mashhad University of Medical Sciences, Mashhad, Iran under ethical code: 931,188. 

### Physical activity and anthropometric assessments

Assessment of physical activity was performed utilizing a modifiable activity questionnaire (MAQ) [28]. Measuring anthropometric parameters such as height, weight, and waist circumference (WC) was performed based on the standard protocols. Calculating body mass index (BMI) was performed using the following formula: weight (kg) divided by square of height (m^2^).

### Dietary assessment

Dietary intakes of participants were collected using a valid and reliable food frequency questionnaire (FFQ), contained 147 food items [29]. Frequency of food items intake during the last year was evaluated by asking the participants about their daily, weekly, monthly and yearly intake. Converting portion sizes to grams was done by household measures. We used the Nutritionist IV software (version 7.0; N-Squared Computing, Salem, OR, USA), modified for Iranian foods, to analyze the dietary intakes [30]. Subjects under- or over-reporting of energy intake (< -3 standard deviation (SD) or > +3SD) were excluded from our analysis.

### Phytochemical index calculation

The DPI score was computed using the following method, that was produced by McCarty [26]: DPI = (daily energy derived from phytochemical-rich foods (kcal)/total daily energy intake (kcal)) × 100. The relationship between DPI and several diseases has been investigated [31–35]. The rich sources of phytochemical are fruits, vegetables, legumes, whole grains, nuts, soy products, seeds and olive oil. The content of phytochemicals in potatoes is not high, and we do not include the potatoes into the DPI formula. Natural fruit and vegetable juices as well as tomato sauces are the rich sources of phytochemicals, were entered in the fruit and vegetable groups.

### Assessment of depression

Assessment of depression was performed using a Persian version of the 21-item beck depression inventory (BDI), that was validated by previous investigations [36, 37]. The items are evaluating various symptoms of depression including feelings of guilt, feelings of hopelessness, sadness, crying, sleep disturbance, fear and loss of appetite over the past 2 weeks. The scores of BDI ranges between 0 (no depression) and 63 (severe depression), and BDI score more than 16 was considered as the cut-off value for the presence of depression [37, 38].

### Quality of life assessment

We assessed the health-related QoL utilizing a validated SF-12v2 questionnaire, which is a short form of the SF-36 questionnaire and improved version of SF-12v1 [39, 40]. The items of SF-12v2 questionnaire are evaluating the domains of health including physical functioning, role limitations because of physical problems, role limitations because of emotional problems, bodily pain, general health, vitality, social functioning, and mental health. The scores of QoL are from 0 to 100, and subjects with scores more than 43 (the median score of QoL is 43) were considered as subjects with high QoL.

### Biochemical assessments

To measure serum concentrations of triglyceride (TG), total cholesterol (TC) and high density lipoprotein-cholesterol (HDL-c), blood samples were obtained while participants were in 14 h overnight fasting. To separate serum and plasma into two aliquots (0.5 mL), blood samples were immediately centrifuged (Hettich model D-78,532) for 10 min at room temperature. Then, serum samples were stored at -80 °C at the reference laboratory in Mashhad University of medical science until analyses. TG, TC and HDL-c were measured by enzymatically method using Pars Azmoon, Karaj, Iran, and the BT-3000 auto-analyzer machine (Biotechnica, Rome, Italy). Low density lipoprotein-cholesterol (LDL-c) was calculated using a validated formula (Friedewald equation) [41].

### Statistical analysis

Utilizing a statistical package for social science (SPSS) software (Chicago, Illinois, USA) version 24, statistical analyses were performed. We categorized the subjects into the four groups based on their DPI scores. General characteristics as well as dietary intakes across quartiles of DPI score were expressed as means ± SDs, and numbers (percentage) for continuous variables and categorical variables, respectively. We used one-way ANOVA for continuous variables and logistic regression analysis for categorical variables to calculate p for trend of each variables across the quartiles of DPI scores. The association between quartiles of DPI scores with depression and poor QoL was evaluated using a logistic regression analysis in the crude and adjusted models. One-way ANOVA for continuous variables (depression score and QoL score) and chi-square test for categorical variables (depression prevalence and poor QoL prevalence) was used to compare the differences between quartiles of DPI scores. In addition, logistic regression in crude and adjusted models was used to compare depression and poor QoL between quartiles of DPI scores. Age and energy intake were adjusted in Model (I) Additionally, BMI was adjusted in Model (II) In the model III, physical activity, age, energy intake, BMI, divorce of parents and death of parents were adjusted. *P* < 0.05 was considered significant.

## Results

### General characteristics study participants

General characteristics of the study participants across categories of DPI are shown in Table 1. The mean age of the participants was 14.5 years. The prevalence of depression and poor QoL were 24.8% and 49.7%, respectively. Systolic blood pressure and diastolic blood pressure significantly increased across quartiles of DPI score (P trend = 0.002). HDL-c significantly decreased (P trend = 0.027) across quartiles of DPI score, but BMI increased (P trend = 0.042). Other variables and general characteristics had no significant trend across quartiles of DPI.

**Table 1:** General characteristics of study participants by quartiles of DPI

Variables	Q1 (*N* = 183 )	Q2 (*N* = 183 )	Q3 (*N* = 184 )	Q4 (*N* = 183 )	P trend*
Dietary phytochemical index (range)	< 15.2	15.2-20.9	20.9-29.9	> 29.9	
Age (year)	14.55 ± 1.52	14.47 ± 1.48	14.49 ± 1.51	14.53 ± 1.63	0.927
BMI (kg/m^2^)	20.76 ± 3.98	20.95 ± 3.78	21.66 ± 4.77	21.47 ± 4.21	0.042
Waist circumference (cm)	69.88 ± 9.03	70.04 ± 8.35	71.41 ± 9.82	70.67 ± 9.10	0.216
WHR	0.76 ± 0.05	0.76 ± 0.07	0.77 ± 0.05	0.76 ± 0.06	0.874
Systolic blood pressure (mm/Hg)	95.26 ± 13.83^b^	95.39 ± 13.69^b^	97.11 ± 14.36	99.56 ± 13.95^a^	0.002
Diastolic blood pressure (mm/Hg)	61.71 ± 14.23^b^	61.68 ± 13.38 ^b^	62.41 ± 13.27	65.98 ± 11.49^a^	0.002
Metabolic equivalent for task (h/week)	45.14 ± 3.11	45.18 ± 3.13	45.35 ± 3.24	45.82 ± 4.17	0.054
Triglyceride (mg/dL)	84.06 ± 38.81	80.81 ± 34.02	85.89 ± 39.08	89.89 ± 46.22	0.657
Total cholesterol (mg/dL)	162.21 ± 27.88	158.66 ± 25.59	165.13 ± 28.32	158.03 ± 30.85	0.946
HDL-c (mg/dL)	46.69 ± 8.92	47.11 ± 8.55	48.31 ± 8.88	45.88 ± 8.06	0.027
LDL-c (mg/dL)	101.28 ± 25.63	94.97 ± 24.04	103.91 ± 26.37	99.11 ± 25.71	0.386
Death of parent (%)	6 (3.4%)	8 (4.5%)	11 (6.1%)	4 (2.2%)	0.786
Divorce of parent (%)	10 (5.6%)	10 (5.6%)	11 (6.1%)	4 (2.3%)	0.193

DPI: dietary phytochemical index. BMI: body mass index. WHR: waist-to-hip ratio. HDL-c: high-density lipoprotein-cholesterol. LDL-c: low-density lipoprotein-cholesterol. SD: standard deviation, values are means ± SD, n (%): numbers (percentage). ^*^Obtained from one-way ANOVA and logistic regression analysis for continuous and categorical variables, respectively. ^a^: last quartile is considered as reference quartile, ^b^: Significant difference between quartile with reference quartile (P value < 0.05).

### Dietary intake study participants

Mean dietary intakes of participants across quartiles of DPI are provided in Table 2. Intake of protein and carbohydrate (per 1000 kcal) across quartiles of DPI significantly increased but fat intake decreased (P trend < 0.001). Vitamin B6, vitamin C, and folate intakes significantly increased across quartiles of DPI (P trend < 0.001), but thiamine intake decreased (P trend = 0.005). Iron and magnesium intakes significantly increased across quartiles of DPI (P trend < 0.001). In addition, intake of saturated fatty acid (P trend < 0.001), MUFA (P trend < 0.001) and PUFA (P trend = 0.005) significantly decreased across quartiles of DPI. Intake of food groups including fruits, vegetables, legumes, nuts and whole grains (per 1000 kcal) significantly increased across quartiles of DPI score (P trend < 0.001). Details are shown in Table 2.

**Table 2:** Dietary intakes of study participants by quartiles of DPI

Variables	**Q1** ***(N = 183)***	**Q2** ***(N = 183)***	**Q3** ***(N = 184)***	**Q4** ***(N = 183)***	P trend^**^
Energy (kcal)*	2751.25 ± 835.61	2619.72 ± 742.90	2793.33 ± 833.42	2868.57 ± 900.95	0.932
Carbohydrate (gr/1000Kcal)	134.62 ± 21.85^b^	135.13 ± 16.88^b^	135.69 ± 16.73^b^	142.83 ± 15.82^a^	< 0.001
Protein (gr/1000Kcal)	32.74 ± 6.21^b^	33.79 ± 5.08^b^	34.32 ± 4.56	35.56 ± 5.91^a^	< 0.001
Fat (gr/1000Kcal)	39.09 ± 10.66^b^	38.15 ± 7.93^b^	36.15 ± 7.58^b^	34.98 ± 7.05^a^	< 0.001
Cholesterol (mg/100Kcal)	86.20 ± 55.66	92.35 ± 39.24	91.64 ± 38.68	87.29 ± 46.17	0.865
Saturated fatty acid (gr/1000Kcal)	11.51 ± 3.82^b^	11.74 ± 3.47 ^b^	11.28 ± 3.06	10.38 ± 2.85^a^	< 0.001
MUFA (gr/1000Kcal)	13.08 ± 4.36^b^	12.13 ± 3.15	12.06 ± 3.08	11.23 ± 2.82^a^	< 0.001
PUFA (gr/1000Kcal)	8.98 ± 3.97^b^	8.20 ± 2.78	8.42 ± 2.73	7.91 ± 2.92 ^a^	0.004
Calcium (mg/1000Kcal)	414.77 ± 150.29	438.53 ± 134.59	439.96 ± 129.99	412.06 ± 120.88	0.880
Iron (mg/1000Kcal)	7.29 ± 1.70^b^	7.12 ± 1.34^b^	7.17 ± 1.21^b^	7.94 ± 1.43^a^	< 0.001
Magnesium (mg/1000Kcal)	174.79 ± 45.60^b^	171.18 ± 35.40^b^	181.52 ± 33.56^b^	206.96 ± 34.39^a^	< 0.001
Thiamine (mg/1000Kcal)	0.89 ± 0.21	0.84 ± 0.16	0.80 ± 0.15	0.85 ± 0.19	0.005
Riboflavin (mg/1000Kcal)	0.81 ± 0.33	0.83 ± 0.19	0.83 ± 0.19	0.81 ± 0.19	0.782
Niacin (mg/1000Kcal)	9.30 ± 2.24	9.05 ± 1.90	8.65 ± 1.82	9.18 ± 2.05	0.242
Vitamin B6 (mg/1000Kcal)	0.68 ± 0.13^b^	0.70 ± 0.11	0.71 ± 0.11	0.74 ± 0.14^a^	< 0.001
Folate (µg/1000Kcal)	214.74 ± 45.11^b^	228.88 ± 44.22	229.98 ± 44.52	239.69 ± 58.66^a^	< 0.001
Vitamin B12 (µg/1000Kcal)	1.75 ± 4.45	1.58 ± 0.71	1.57 ± 0.67	1.44 ± 0.77	0.221
Vitamin C (mg/1000Kcal)	24.89 ± 11.32^b^	33.48 ± 15.09^b^	41.33 ± 19.83	42.86 ± 27.21^a^	< 0.001
Vitamin A (IU/1000Kcal)	209.25 ± 438.47	216.99 ± 98.04	231.29 ± 100.13	239.44 ± 138.68	0.188
Fruits (gr/1000 kcal)	48.90 ± 29.42^b^	79.24 ± 43.83^b^	105.45 ± 70.05	113.86 ± 97.87^a^	< 0.001
Vegetables (gr/1000 kcal)	65.98 ± 45.88^b^	83.30 ± 48.62^b^	98.70 ± 53.53	110.56 ± 80.14^a^	< 0.001
Legumes (gr/1000 kcal)	15.59 ± 9.77^b^	24.86 ± 15.80^b^	29.59 ± 16.08	33.57 ± 27.96^a^	< 0.001
Nuts and seeds (gr/1000 kcal)	2.00 ± 1.88^b^	3.83 ± 3.65^b^	6.72 ± 6.43^b^	10.44 ± 15.04^a^	< 0.001
Whole grains (gr/1000 kcal)	3.69 ± 5.02^b^	8.91 ± 10.70^b^	13.80 ± 17.02^b^	57.36 ± 52.62^a^	< 0.001

*Values are means ± SDs and adjusted for energy intake,^**^ Obtained from one-way ANOVA; DPI: dietary phytochemical index. MUFA: monounsaturated fatty acid. PUFA: polyunsaturated fatty acid. ^a^: last quartile is considered as reference quartile, ^b^: Significant difference between quartile with reference quartile (P value < 0.05).

### The association between DPI with depression and poor QoL

The prevalence of depression significantly decreased across quartiles of DPI score (P trend = 0.009). In addition, a significant reduction in depression score was found across quartiles of DPI score (P trend = 0.002). Adolescents in the highest quartile of DPI compared with the first quartile had a 50% lower odds of depression (OR: 0.50; 95% CI: 0.30–0.84, *P* = 0.009). This relation remained significant after adjustment for age, energy intake, BMI, physical activity, divorce of parents and death of parents (OR: 0.48; 95% CI: 0.28–0.83, *P* = 0.008). In all models, trend for odds of depression significantly reduced across DPI score (Table 3).

**Table 3:** The prevalence, score and OR (95%CI) of depression and poor QoL by quartiles of DPI

		Quartiles of DPI				P value*	P trend**
		**Q1**	**Q2**	**Q3**	**Q4**		
**Depression score**		12 ± 9.59	11.82 ± 9.63	10.65 ± 9.10	9.22 ± 8.55	0.015^a^	0.002^a^
**Depression prevalence**		51 (27.9%) Std. Residual = -0.5	53 (29%) Std. Residual = -0.6	48 (26.1%) Std. Residual = -0.2	30 (16.4%) Std. Residual = 1.3	0.021^b^	0.009^c^
**Depression**	Crude	1.00	1.05 (0.67-1.66)	0.91 (0.57-1.44)	0.50 (0.30-0.84)	0.009^c^	0.009^c^
	Model1	1.00	1.04 (0.66-1.64)	0.91 (0.57-1.45)	0.50 (0.30-0.83)	0.008^c^	0.009^c^
	Model2	1.00	1.04 (0.66-1.65)	0.93 (0.58-1.48)	0.51 (0.30-0.85)	0.010^c^	0.011^c^
	Model3	1.00	1.00 (0.63-1.60)	0.84 (0.52-1.36)	0.48 (0.28-0.83)	0.008^c^	0.008^c^
**QoL score**		41.60 ± 8.20	41.37 ± 7.76	42.42 ± 8.28	42.87 ± 7.62	0.24^a^	0.067^a^
**Poor QoL prevalence**		98 (54.1%) Std. Residual = -0.8	100 (55.6%) Std. Residual = -1.1	86 (47%) Std. Residual = 0.5	77 (42.3%) Std. Residual = 1.4	0.038^b^	0.008^c^
**Poor QoL**	Crude	1.00	1.05 (0.69-1.60)	0.75 (0.49-1.13)	0.62 (0.41-94)	0.024^c^	0.008^c^
	Model1	1.00	1.05 (0.69-1.60)	0.75 (0.49-1.13)	0.62 (0.40-0.93)	0.024^c^	0.008^c^
	Model2	1.00	1.06 (0.70-1.61)	0.77 (0.51-1.16)	0.63 (0.41-0.95)	0.030^c^	0.011^c^
	Model3	1.00	1.11 (0.73-1.70)	0.77 (0.51-1.18)	0.67 (0.43-1.02)	0.066^c^	0.023^c^

*Fourth quartile compared to first quartile.

**Trends across DPI quartiles.

^a^: Obtained from one-way ANOVA

^b^: Obtained from chi-square

^c^: Obtained from logistic regression

Model I: Adjusted for age and energy intake

Model II: Additionally, adjusted for BMI.

Model III: Additionally, adjusted for physical activity, divorce of parents and death of parents.

OR: odds ratio. CI: confidence interval. DPI: dietary phytochemical index. QoL: quality of life. BMI: body mass index.

We found a significant reduction in prevalence of poor QoL across quartiles of DPI score (P trend = 0.008). However, no significant reduction in QoL score was observed across quartiles of DPI score (P trend = 0.067). The participants in the highest quartile of DPI compared with the lowest quartile had a 38% lower odds of poor QoL (OR: 0.62; 95% CI: 0.41-0.94, *P* = 0.02). This association was not significant after adjustment for age, energy intake, BMI, physical activity, divorce of parents and death of parents (OR: 0.67; 95% CI: 0.43–1.02, *P* = 0.06). In all models, trend for odds of poor QoL significantly reduced across DPI score (Table 3).

## Discussion

Depression is an important precipitating cause of suicide ideation and suicide attempts in adolescents; therefore, management of depression is necessary [5, 6]. Diet as a modifiable lifestyle factor has an important role in depression and subsequent QoL [42, 43]. Some studies revealed that adherence to the healthy, plant-based dietary patterns is inversely associated with risk of mental disorders [17, 44]. Intake of plant foods that are rich sources of phytochemicals can increase the serum levels of phytochemicals [45]. For example, it has been confirmed that consuming whole grains, vegetables, whole fruit, legumes, nuts, seeds is directly associated with serum phytochemicals [45]. In addition, serum phytochemicals are reliable variables of fruits and vegetables intake [46]. It has been reported that DPI, which is based on compounds with antioxidant properties, is directly associated with total carotenoid intake and is inversely associated with levels of oxidative stress [47]. This finding can confirm the accuracy of DPI in estimating phytochemicals intake. To our knowledge, the present cross-sectional study is the first study investigating the relationship between DPI with depression and QoL in adolescent females.

We demonstrated that higher DPI score is associated with decreased risk of depression. Only one cross-sectional study examined the association between DPI and mental disorders among adults [4], and demonstrated an inverse relationship between DPI scores with the risk of depressive symptoms, anxiety and psychological stress. Findings from a cohort study suggested that higher intake of flavonoids is associated with lower risk of incident depression [48]. In addition, the Mediterranean healthy eating, aging and lifestyle (MEAL) study reported an inverse relationship between intake of polyphenols and risk of depression [49]. Generally, phytochemicals by regulating dopaminergic brain pathways, enhancing serotonin and dopamine levels in the hippocampus and prefrontal cortex, and reducing monoamine oxidase (MAO) activity may improve depressive symptoms [19–21, 50].

On the other hand, a cross-sectional study revealed that higher intakes of fruits and vegetables are associated with levels of oxidative stress and inflammation as the main contributors of the pathogenesis of depression [51]. In addition, some studies have demonstrated that phytochemicals can improve oxidative stress [52, 53] and inflammation [54–56]. Phytochemicals can suppress fatty acid synthesis and gluconeogenesis, increase mitochondrial oxidation, reduce the levels of oxidant agents as well as free radicals, and attenuate the activity of inflammatory markers [22–24, 57, 58].

There was no significant relationship between DPI score and QoL after adjusting for all confounding factors (Model III). However, we found a significant reduction trend in the odds of poor QoL across increasing quartile of DPI score. The relationship between DPI score with poor QoL remained unclear. Therefore, the findings in this field should be interpreted with caution. There was no investigation evaluating the association of phytochemicals with QoL. It has been shown that greater adherence to the Mediterranean diet as a plant-based diet, is associated with better QoL [59, 60]. In addition, higher quality diets containing higher amounts of whole grains, fruits and vegetables as well as lower amounts refined foods and fast food are associated with better QoL [61–63]. Some food groups such as dairy products and seafood, which are found in these healthy dietary patterns, are not the sources of phytochemical, and we did not use these food groups in the calculation of DPI; therefore, specific investigations evaluating the association between phytochemicals and QoL are required.

The present study has some important strengths. This is the first study investigating the relationship between DPI scores depression and QoL among adolescents. In addition, we controlled the effects of several confounding factors. This study has some limitations. This is a cross-sectional study and we cannot show the causal link between DPI with depression and QoL. In addition, we did not include adolescent males in the study. Moreover, green and black tea (which are the rich sources of phytochemicals) were not considered for DPI calculation, because DPI formula is based on energy of phytochemicals sources and green and black tea did not contribute energy intake. Furthermore, we used FFQ to assess dietary intakes of participants, but dietary weighing method has higher accuracy compared to the FFQ.

In conclusion, we found that DPI score is negatively associated with risk of depression. However, according to inconsistent results, the associations between DPI score with poor QoL remained unclear. Further studies should be conducted, especially on both females and males. In addition, prospective and interventional investigations are needed to clarify casual relationships.

## Data Availability

The data and materials of the current study is available from the corresponding author on reasonable request.

## Abbreviations

BDI: beck depression inventory
BMI: body mass index
DPI: dietary phytochemical index
FFQ: food frequency questionnaire
HDL-c: high density lipoprotein-cholesterol
LDL-c: low density lipoprotein-cholesterol
MAO: monoamine oxidase
MAQ: modifiable activity questionnaire
MEAL: Mediterranean healthy eating, aging and lifestyle
OR: odds ratio
QoL: quality of life
SD: standard deviation
SPSS: statistical package for social science
TC: total cholesterol
TG: triglyceride
WC: waist circumference
